# Inflammation Markers in Adipose Tissue and Cardiovascular Risk Reduction by Pomegranate Juice in Obesity Induced by a Hypercaloric Diet in Wistar Rats

**DOI:** 10.3390/nu13082577

**Published:** 2021-07-27

**Authors:** Maria Monica Michicotl-Meneses, María del Rocío Thompson-Bonilla, César A. Reyes-López, Blanca Estela García-Pérez, Itzel I. López-Tenorio, Cynthia Ordaz-Pichardo, María Eugenia Jaramillo-Flores

**Affiliations:** 1 Departamento de Ingeniería Bioquímica, Instituto Politécnico Nacional, Escuela Nacional de Ciencias Biológicas, Mexico City 07738, Mexico; michicotlm4@gmail.com (M.M.M.-M.); itzeltenorio20@hotmail.com (I.I.L.-T.); 2 Laboratorio de Medicina Genómica, Investigación Biomédica y Traslacional, ISSSTE, Hospital Regional “1° de Octubre”, Mexico City 07760, Mexico; rociothompson@yahoo.com.mx; 3 Laboratorio de Bioquímica Estructural, Instituto Politécnico Nacional, Escuela Nacional de Medicina y Homeopatía, Mexico City 07320, Mexico; careyes@ipn.mx; 4 Laboratorio de Microbiología General, Instituto Politécnico Nacional, Escuela Nacional de Ciencias Biológicas, Mexico City 11340, Mexico; abrilestela@hotmail.com; 5 Laboratorio de Biología Celular y Productos Naturales, Instituto Politécnico Nacional, Escuela Nacional de Medicina y Homeopatía, Mexico City 07320, Mexico; dra_cynthia@hotmail.com

**Keywords:** pomegranate juice, anthocyanins, obesity, pro-inflammatory cytokines, inflammation, selectin, atherosclerosis vascular risk, high-fat diet

## Abstract

Pomegranate juice (*Punica granatum*) has been used since ancient times in traditional medicine (Unani Medicine, Ayurveda); its main compounds are anthocyanins and ellagic acid, which have anti-inflammatory, antioxidant, hepatoprotective, and cardiovascular health effects. The objective was to evaluate the effect of pomegranate juice on inflammation, blood pressure, and vascular and physiological markers associated with obesity induced by a high-fat diet in a murine model. The results show that pomegranate juice reduces the concentration of low-density lipoprotein cholesterol (cLDL) 39% and increases the concentration of high-density lipoprotein cholesterol (cHDL) by 27%, leading to a 12%–18% decrease in the risk of cardiovascular diseases (CVD). In addition to reducing blood pressure by 24%, it also had an antiatherogenic effect by decreasing sE-selectin levels by 42%. On the other hand, the juice significantly increased adiponectin levels in adipose tissue, decreased levels of inflammation markers (tumor necrosis factor-α (TNF-α), plasminogen activator inhibitor-1 (PAI-1), interleukin-17A (IL-17A), interleukin-6 (IL-6), interleukin-1β (IL-1β)), and inhibited the monocyte chemoattractant protein-1 (MCP-1). Pomegranate juice requires clinical studies to prove its immunoregulatory and therapeutic effects on cardiovascular and atherogenic risks.

## 1. Introduction

Metabolic diseases, including obesity, are one of the main public health problems. Obesity is the excessive accumulation of visceral and subcutaneous fat from adipose tissue (AT), and this accumulation is strongly associated with alterations in lipid metabolism, endothelial dysfunction, insulin resistance, dysregulation of adipokines expression, and inflammation. These changes are considered a risk factor for developing metabolic syndrome (MS), type 2 diabetes (T2DM), cardiovascular disease (CVD), non-alcoholic fatty liver (NAFLD), endothelial damage, atherosclerosis, and hypertension [1,2]. All of these diseases contribute to the high mortality and morbidity rates prevalent worldwide.

In recent years, strong evidence has highlighted the main role of inflammation as the mechanism responsible for making obesity a risk factor in developing these metabolic complications [3,4]. The inflammation of adipose tissue promotes the loss of the immune regulation and favors the infiltration and activation of immune cells, which in turn, produce increased levels of pro-inflammatory cytokines, such as interleukin 6 (IL-6), interleukin 1α (IL-1α), interleukin 1β (IL-1β), tumor necrosis factor (TNF-α), plasminogen activator inhibitor-1 (PAI-1), and monocyte chemoattractant protein-1 (MCP-1). Moreover, the persistence of M1-polarized macrophages contributes to maintenance of the low-grade chronic inflammation in obesity [5]. Moreover, the dysfunction of adipose tissue leads to a dysregulated production in the levels of anti-inflammatory adipokines (adiponectin) [6]. Obesity is also associated with endothelial dysfunction and stimulation of the proliferative response in the vascular wall, thus promoting an increased risk of cardiovascular diseases (CVD), such as dyslipidemia, thrombosis, atherogenesis, atherosclerosis, and hypertension [7]. Endothelial dysfunction is generated by exposure to an activating stimulus, for instance, proinflammatory cytokine secretion, triglyceride elevation, decreased high-density lipoprotein cholesterol (HDL-C), and increased low-density lipoprotein cholesterol (LDL-C) [7,8], which induce the expression of cell adhesion molecules on the endothelial surface, such as vascular cell adhesion molecule 1 (VCAM -1), the intercellular adhesion molecule 1 (ICAM-1), and E-selectin, that participate in the inflammatory reaction, as well as high levels of nitric oxide (NO) produced by inducible nitric oxide synthase (iNOS), ultimately promoting atherosclerosis [9]. The role of anti-inflammatory cytokines, such as adiponectin secreted by adipose tissue (whose levels in obese patients are decreased compared to healthy people), is to improve insulin sensitivity by increasing energy expenditure and the oxidation of fatty acids through the phosphorylation of protein kinase (AMPK) activated by 5-adenosine monophosphate (5-AMP) through increased expression of Peroxisomal Proliferators-Activated Receptors (PPAR’s), Cluster of Differentiation 36 (CD36), acyl-coenzyme oxidase, and uncoupling protein 2 (UCP2) genes [10]. On the other hand, its vasculoprotective function has been demonstrated since it attenuates monocyte adhesion to endothelial cells, mainly by the inhibition of TNF-α and the synthesis of intercellular adhesion molecule 1 (ICAM-1), the molecule of cell adhesion 1 (VCAM 1), and E-selectin; therefore, adiponectin limits the onset of atherosclerotic plaque [11].

Since the collected evidence indicates that a state of chronic inflammation has a crucial role in the pathogenesis of obesity-related metabolic dysfunction, where clinical and epidemiological studies describe a clear connection between high levels of proinflammatory cytokines with atherosclerotic lesions that favor cardiovascular events [12], several efforts to integrate interventions targeting lowering the inflammation burden have been carried out [13].

Pomegranate (*Punica granatum*) is an edible fruit whose nutraceutical and pharmacological properties have been reported [14,15]. Its main antioxidant compounds are hydrolysable tannins, anthocyanins (3-glycosides and 3,5-glycosides of delphinidin, cyanidin, and pelargonidin) and derivatives of ellagic acid. There are some differences in the composition of phenolic compounds between processed and natural juices, due to, among others, the type of process, cultivation, and growing conditions [16,17]. Studies in cell cultures, mice, and humans show the effect of flavonoids present in the juice, which are associated with the prevention of cardiovascular diseases, obesity, diabetes, and cancer [18].

The consumption of natural or concentrated pomegranate juice has beneficial effects on human health, since prolonged supplementation, in vitro and in vivo, with pomegranate juice concentrate and pomegranate fruit extract ameliorated the perturbed shear stress-induced atherogenesis by increasing the activity of endothelial nitric oxide synthase (eNOS) and reducing the protein levels of oxidation-sensitive responsive genes (ELK-1 and pCREB) [19]. Consuming 200 mL/day of pomegranate juice for 6 weeks in patients with T2DM decreased systolic pressure and diastolic pressure and improved the lipid profile, reducing total cholesterol and LDL-c without effect on HDL-c [20]. Additionally, daily supplementation with 20 g of microencapsulated pomegranate for 30 days reverses endothelial dysfunction and reduces postprandial triglyceridemia in women with acute coronary syndrome [21]. Therefore, the objective of this study was to evaluate the effect of pomegranate juice on the markers of inflammation (TNF-α, IL-1β, IL-6, MCP-1, and PAI-1) in retroperitoneal adipose tissue, which is associated with markers of vascular disorders (E-selectin, cHDL y cLDL) and metabolic alterations in obesity.

## 2. Materials and Methods

### 2.1. Study Material

The fruit of ripe *Punica granatum* Tecozautla cultivar from the San Nicolas de los Ranchos and Huejotzingo, Puebla, Mexico, was used, which is characterized as a fruit of thin yellowish green skin, dark red, sweet aryl, and semi-hard seed.

### 2.2. Obtaining the Pomegranate Juice

Fresh pomegranate fruits were selected from the *Punica granatum* Tecozautla cultivar for their physical and sensory characteristics, indicating they were appropriate to be consumed, such as the external color light red and greenish yellow; internal color: red; flavor: sweet and sour, sweet predominates; appearance: globular with 10 cm wide without physical defects; and free of pests and diseases (CXS 310-2013). The fruits were washed to extract manually the peel, endocarp, and aryls; they were stored at −80 °C for later use. The aryls were pressed manually to obtain the juice, which was stored in an amber bottle at −80 °C for later use.

### 2.3. Quantification of Total Anthocyanins

The differential pH method was used [22]. The concentration of total monomeric anthocyanins was determined as follows: in a test tube, 1 mL of the sample and 7 mL of a potassium chloride buffer (pH 1.0) were placed; in another tube, 1 mL of the sample and 7 mL of a sodium acetate buffer (pH 4.5) were placed, allowing them to react in the dark for 15 min. Absorbance readings at 510 nm and 700 nm were taken in a UV-Visible Spectrophotometer (Genesys 10S UV-Vis, Thermo Scientific, Madison, WI, USA). The results were expressed as mg of cyanidin-3-glycoside equivalent per kilogram sample (C_3_g Eq/Kg of juice).

The calculations were carried out using the following equations:

A = (Abs_510_ − Abs_700_) _pH 1_._0_ − (Abs_510_ − Abs_700_) _pH 4_._5_

$$\mathrm{Concentration}\;\mathrm{Monomeric}\;\mathrm{Anthocyanins}\;(\mathrm{MAC})=\frac{\left(A\times \mathrm{MW}\times \mathrm{FD}\right)}{\varepsilon \times L}\times 100$$

where: A = absorbance; ε = molar absorptivity cyanidin-3-glucoside (26.900); MW = molecular weight cyanidin-3-glucoside (449.2 g/mol); FD = dilution factor; L = cell length.

### 2.4. Determination of Total Phenols

The phenolic compounds of the juice were determined by the method described by Herald et al. [23]; a SINERGY H1 microplate reader was used (Biotek, Winoosky, VT, USA). In a 96-well plate, 75 µL of distilled water was added followed by 25 µL of the sample or standard and 25 µL of Folin–Ciocalteu reagent (1:1); they were allowed to react for 6 min, and then 100 µL of Na_2_CO_3_ was added (75 g/L). The plate was covered and kept in the dark for 90 min. The absorbance was measured at 750 nm in triplicate using gallic acid as a standard at concentrations between 12.5 and 200 µg/mL. The results obtained are expressed as mg of gallic acid (AG) Eq/g of sample.

#### Phenol Compounds Identification

The identification of phenolic compounds in pomegranate juice was carried out by reversed phase HPLC on an Agilent 1260 Infinity^®^ HPLC (Agilent Technologies, Santa Clara, CA, USA) with a quaternary pump, automatic injector and detector of diode array (HPLC-DAD), HPLC DAD software, and Zorbax C-18 column with an internal diameter of 150 × 4.6 mm 0.5 μm (Agilent Technologies Inc. Santa Clara, CA, USA) with injection volume 10 μL and flow 0.5 mL/min, as reported in [24].

### 2.5. Animal Testing

For the study, 6-week-old male Wistar rats (180 ± 10 g body weight) acquired from UPEAL CINVESTAV-IPN (Zacatenco, CDMX, Mexico City, Mexico) were used. All animals received food and water ad libitum and were housed in stainless steel cages with sterile beds under controlled conditions of temperature (22 ± 2 °C) and relative humidity (50 ± 10%) with light–dark cycles of 12 h (9:00–21:00) [21]. The treatment and euthanasia were carried out in accordance with the approval of the BioEthics Committee of the Escuela Nacional de Medicina y Homeopatía CBE/005/2021, complying with the guide for the care, use, and humanitarian sacrifice of the laboratory Animals of the Mexican Animal Care Council (Consejo Mexicano del Cuidado Animal) (NOM-033-ZOO-1995, NOM-062-ZOO-1999) and guidelines of the International Committee. The animals were randomly distributed in 3 groups, standard diet (SD), high-fat diet (HD), and high-fat diet plus pomegranate juice (JP), with an *n* = 6 for each group.

The animals were subjected to an acclimatization period of 1 week prior to the start of the study, which lasted 8 weeks. The SD group was fed with a standard diet (Rodent Laboratory Chow 5001; PMI Nutrition International, St. Louis, MO, USA) and the high-fat diet (HD) and pomegranate juice (JP) groups were fed a high-fat (HD) diet (TD. 88137; Teklad Global Harlan Laboratories, Inc.). The nutritional composition of the standard diet was: 23% of total protein; 46.5% of carbohydrates and 4.5% of fat, corresponding to an energy value of 3.1 kcal/g; and the composition of the high fat diet was: 15.2% protein, 42.8% carbohydrates, and 42% fat, containing 195 g/kg of casein, 3 g/kg of DL-methionine, 340 g/kg of sucrose, 140.5 g/kg of cornstarch, 210 g/kg of butter, 50 g/kg of cellulose, 43 g/kg of mineral MIX, 15 g/kg of vitamin MIX, 2 g/kg of choline, 1.5 g/kg of cholesterol, and an energy content of 4.5 Kcal/kg. The SD and HD groups were given water as a placebo, and the JP group was given pomegranate juice as treatment (10 mL/Kg of body weight daily) intragastrically.

### 2.6. Food and Energy Consumption and Body Weight

The average weekly food consumption was calculated, and caloric intake was determined by calculating food consumption multiplied by dietary energy density.

The animals were weighed at the beginning of the treatment, monitoring the body weight of each rat 3 times per week during the study period.

### 2.7. Adipose Tissue Extraction

At the end of the study period (8 weeks), prior to the administration of ketamine and xylazine (35 and 5 mg/kg) for the euthanasia of the animals, they were subjected to a 12 h fast. Retroperitoneal and epididymal adipose tissue were obtained, which were washed with a 1× phosphate saline (PBS), weighed and frozen immediately, and stored at −80 °C for later use.

### 2.8. Insulin Tolerance

The insulin tolerance test was performed at week 8 of the treatment; the animals were subjected to a 4 h fast, taking blood samples by tail puncture to determine the level of blood at time zero to baseline measures. Subsequently, 0.06 IU/Kg body weight of recombinant human insulin (Humulin R, from Eli Lilly, Mexico City, Mexico) was administered to determine if they exhibited insulin resistance (IR); blood samples were taken after injection intraperitoneally at 30, 60, 90, 120, and 150 min. Glucose levels were determined by using a glucometer (ACCU-CHEK^®^ Performa, Roche Diagnostic, Indianopolis, IN, USA) [25].

### 2.9. Determining Systolic and Diastolic Blood Pressure

The pressure was measured at the end of week 7 of the treatment by the method of tail-cuff plethysmography (non-invasive method) with a pressure gauge Brand IITC Life Science model MRBP System. The animals were placed in a containment device (sepo) for measurement purposes with a noise-free environment prior to the 1-week training until the reading [26].

### 2.10. Lipid Profile

Serum triglyceride and total cholesterol levels were determined by using the RANDOX Kit: total cholesterol RX MONZA CH 200, Triglycerides RX MONZA TR 210, HDL RX SUZUKA CH 8033 (Randox Laboratories Limited, Crumlin, UK) in a SELECTRA II VITA Lab device from Wiener Lab. Blood samples were taken from the abdominal aorta in BD Vacutainer^TM^ SST tubes, which were centrifuged at 2325× *g* for 15 min at 4 °C to obtain the serum. The determinations were made in triplicate. The methodology was performed according to the kit protocol.

### 2.11. Determining Vascular Markers

The levels of sE-selectin, Intercellular adhesion molecule (sICAM), von Willebrand Factor (VWF), and adiponectin were determined in the serum by using the MILLIPLEX^®^ MAP Rat Vascular Injury Magnetic Bead Panel 2 Toxicity Multiplex RVMAG-26K-04 Commercial Kit, which was purchased with MILLIPORE (Billerica, MA, USA). The serum was obtained by taking abdominal aortic blood in BD Vacutainer^TM^ SST tubes, which were centrifuged at 2325× *g* for 15 min at 4 °C. The determinations were made in triplicate. The MAGPIX^®^ Luminex device was used. The methodology was performed according to the kit’s protocol.

### 2.12. Determining Inflammation Markers

Retroperitoneal adipose tissue (100 mg) was homogenized in 700 µL of an extraction buffer for the RIPA lysis assay (50 mM NaH_2_PO_4_, 100 mM Na_2_HPO_4_, 0.1% sodium dodecyl sulfate, 0.5% NaCl, 1% Triton X-100, 1 mM EDTA), and they were frozen with liquid nitrogen until use. Then, they were thawed and centrifuged at 2325× *g* at 4 °C to obtain the supernatant and determine Leptin, TNFα, PAI-1, Insulin, IL1-α, IL-1β, IL-6, and IL-10 levels. The concentrations of Leptin, TNFα, PAI-1, and Insulin were determined by immunoassay with commercial kit MILLIPLEX^®^ MAP Rat Adipokine Panel-Metabolism Assay RADPKMAG-80K (MILLIPORE), and IL1-α, IL-1β, IL-6, and IL-10 levels were determined by commercial kit MILLIPLEX MAP Rat Cytokine/Chemokine Magnetic Bead Panel-Immunology Multiplex Assay (MILLIPORE). The MAGPIX^®^ Luminex equipment was used; all determinations were performed in triplicate, and the results were expressed in pg/g adipose tissue (AT).

### 2.13. Statistical Analysis

The results were previously subjected to normality tests in order to define the statistical test for analysis. The results are expressed as mean values ± standard error. Statistical analysis was determined by a one-way ANOVA, and comparisons between the groups were evaluated by using the Bonferroni or Tukey test for multiple comparisons with a significance level of α ≤ 0.05. The results are expressed as mean values ± standard error. Statistical analysis was determined by non-parametric tests; comparisons between groups were evaluated by using the Mann–Whitney–Wilcoxon test with a level of significance of α < 0.05.

## 3. Results and Discussion

### 3.1. Chemical Composition, Phenolic Compounds

The content of total phenolic compounds and anthocyanins present in the juice were 6.17 mg AG Eq/mL and 0.191 mg C3g Eq/mL, respectively, compared to the content of phenolic compounds of the juice of 10 pomegranate cultivars in four regions of China, where the concentration of polyphenols and anthocyanins ranged between 3.15 and 7.43 mg GaE/mL and between 0.004 and 0.160 mg CyE/mL, respectively [24]. The maximum concentrations found in both groups of compounds are very similar to those of this work, only with a difference of 1.2 mg GaE/mL in phenols and 31 mg of anthocyanins. In contrast, different extraction procedures were compared, in which they found no significant differences between them, showing contents for total phenols of 1.38 to 2.89 mg GaE/mL, and of anthocyanins between 0.109 and 0.139 mg C3gE/mL [27], much lower values than those reported here. The results of the phenol content of this study are two times more than those reported by Mphahlele et al. (2016), whereas in the anthocyanin content, there is no difference.

Regarding the content of phenolic compounds and anthocyanins in pomegranate juice, it has been reported that it contains from 6 to 65 different anthocyanins, some evaluated by HPLC and others by HPLC-MS, which explains the great difference, while in relation to phenolic compounds, up to 86 are reported, which include those from phenolic acids, flavonoids, and ellagitannins and others not identified. Within this large number of bioactive compounds of a phenolic nature, 35 of them are the main ones, highlighting pelargonidine-3,5-diglucoside, cyanidine-3-glucoside, catechin gallato, catechin, and, in lower concentrations, punicalagin α and β [28,29,30,31,32]. In the present work, punicalagin, catechin, chlorogenic acid, gallic acid, ferulic acid, caffeic acid, and ellagic acid were identified by HPLC-DAD.

### 3.2. Effect of Pomegranate Juice on Physiological Parameters

The results of the physiological and biochemical markers obtained in the animal model are shown in Table 1. It is observed that the total body weight gain was greater for the HD control group, with 312.5 g, while the group treated with pomegranate juice (JP) had 25% less weight, since it only reached 234.6 g, even though both groups consumed a high-fat diet. With a balanced diet, it is possible to maintain a normal weight, which hardly happens with a high calorie intake. Therefore, the effect of the JP treatment is relevant, since it reduces weight and adiposity; this can be seen in the retroperitoneal adipose tissue that was reduced by 15%. Since ellagic acid inhibits adipogenesis by modifying chromatin in preadipocytes, thereby reducing adiposity [33], the reduction in adipose tissue is likely due in part to the ellagic acid.

The weight gain is a consequence of the amount of food consumption and the corresponding energy intake. The HD group increased their weight by consuming 25 g/day of food with an energy ingested of 113.5 Kcal/day of a hypercaloric diet, unlike the JP group that had a decrease in food consumption of 7% and energy ingested of 107 Kcal/day. These results suggest that the effect of pomegranate juice on weight reduction in animals with a hypercaloric diet may be due to a decreased appetite. The appetite suppressant effect of pomegranate juice may be due to an inhibitory effect on the production of ghrelin, a gastric hormone that regulates appetite [34], or its potential ability to regulate leptin levels, so that it prevents hyperleptinemia [33,34]. After food intake, leptin levels doubled in obese rats compared to healthy rats, with 4 ng/mL and 2 ng/mL, respectively, while after 24 h of fasting, the reported values were 2 ng/mL for obese rats and 1 ng/mL for healthy rats [35].

**Table 1:** Physiology parameters evaluated in the murine study model.

Parameters	Group SD			Group HD			Group JP		
Food consumption (g/day)	28.21 ^a^	±	0.34	25.37 ^b^	±	0.80	23.72 ^c^	±	0.16
Energy consumption (Kcal/day)	87.45 ^a^	±	1.07	113.5 ^b^	±	3.87	106.78 ^c^	±	0.70
Gain in body weight (g)	212.6 ^a^	±	4.26	312.5 ^b^	±	8.13	234.62 ^a^	±	7.30
Retroperitoneal adipose tissue (%)	1.9 ^a^	±	0.03	3.8 ^b^	±	0.12	3.24 ^c^	±	0.09
Epididymal adipose tissue (%)	1.48 ^a^	±	0.04	2.59 ^b^	±	0.18	2.67 ^b^	±	0.08
Glucose (mg/dL)	112.3 ^a^	±	0.97	128.2 ^b^	±	3.49	115.5 ^a^	±	2.95
Systolic pressure (mmHg)	117.7 ^a^	±	1.26	141.9 ^b^	±	1.68	112.61 ^a^	±	0.16
Diastolic pressure (mmHg)	90.33 ^a^	±	2.67	116.7 ^b^	±	2.46	88.89 ^a^	±	2.69

SD: standard diet; HD: high-fat diet; JP: high-fat diet + pomegranate juice. Values represent the mean ± standard error. Statistical analysis was determined by using a one-way ANOVA followed by the Bonferroni test for multiple comparisons with a level of significance of *p* ≤ 0.05. Different letters (^a^, ^b^ and ^c^) indicate statistical significance between the groups (columns).

The excessive intake of a high-fat diet triggers the deregulation of body weight generating obesity, associated with endocrine and metabolic changes. This behavior was reflected in the HD control group with 3.8% retroperitoneal adipose tissue and 2.6% epididymal adipose tissue, which significantly increases the risk of cardiovascular complications, NAFLD, insulin resistance, atherosclerosis, dyslipidemia, and T2DM. The JP treatment suggests an anti-obesity effect, since it reduces the retroperitoneal adipose tissue by 15% with respect to the HD, although without effect on the epididymal adipose tissue.

Intra-abdominal adipose tissue (retroperitoneal and peritoneal) [36] contributes to the appearance of metabolic disorders, such as glucose dysregulation, generating hyperglycemia that is associated with insulin resistance [37,38]. In humans, ellagic acid has been reported to reduce blood glucose levels [39].

Regarding blood pressure, the results obtained showed that the treatment decreased systolic pressure from 142 to 112.61 mmHg and reduced the diastolic pressure from 116.7 to 88.89 mmHg, with the blood pressure values reaching those of the SD group. These results indicate that pomegranate juice can regulate blood pressure to normal levels (100–120 mmHg in systolic pressure and 84–90 mmHg in diastolic pressure) [40,41,42]. Ellagic acid, one of the main components of pomegranate juice, reduces blood pressure in hypertensive rats, probably improving the bioavailability of nitric oxide [43], as well as catechin and ferulic acid, the latter attenuating oxidative stress [44,45]. Epidemiological studies have shown that blood pressure and obesity are risks for cardiovascular disease [39]. A clear association of weight gain with hypertension has been demonstrated, since 60% of hypertension is attributed to the increase in adipose tissue stores, where obese people are 3.5 times more likely to have hypertension compared to healthy individuals. Data from the National Health and Nutrition Examination Survey (NHANES) indicate that the prevalence of hypertension among obese people with a body mass index (BMI) > 30 kg/m^2^ is 42.5% compared to 15.3% for thin people [46]. They studied in Wistar rats with diabetes and hypertension induced with Streptozotocin and Angiotensin II (STZ and Ang II) the effect of dehydrated and concentrated pomegranate juice under reduced pressure (pomegranate juice extract) in doses of 100 and 300 mg/Kg/ day for 4 weeks. They observed a 36% reduction in blood pressure with respect to diabetic and hypertensive groups, but without difference with the control group (healthy), indicating beneficial effects of the pomegranate juice extract on blood pressure in both diabetes and hypertension [47]. A decrease in blood pressure was obtained in this study, and therefore the risk of suffering from cardiovascular diseases in obese patients, agreeing with the previously mentioned studies [48]; this is probably due to the anthocyanins and ellagic acid present in pomegranate juice [43,44].

### 3.3. Lipid Profile

The levels of total cholesterol, triglycerides, c-HDL, and c-LDL (Table 2) showed that the levels of total cholesterol were not modified, while the ratio of cHDL-cLDL was favorably modified, such that cHDL 5.87 mg/dL was increased and LDL-C decreased by 6.3 mg/dL with treatment. Furthermore, there was a slight increase (7%) in triacylglycerides; this is probably due to the sugar content of the juice. Elevated levels of c-HDL have been associated with the reduction in cardiovascular disease risks [45].

Experimental evidence in humans and animal models confirms that increased levels of c-HDL reduces atherosclerosis, since every 1 mg/dL (0.0259 mmol/L) of increased c-HDL equals a 2%–3% reduced risk of cardiovascular diseases (CVD) [49], which would be equivalent to a risk reduction of between 12%–18% with this treatment. The anthocyanins present in the juice suggest a protective effect to risks of atherosclerosis and cardiovascular diseases. Likewise, preclinical and clinical studies have evaluated the bioactivity of dietary anthocyanin intake, and it is associated with beneficial changes in serum biomarkers in human populations (hyperlipidemic, hypertensive, or diabetic) that include not only the levels of cHDL, but also its anti-inflammatory, antioxidant, vasodilator, and/or antithrombotic properties. Therefore, it has been proposed that anthocyanins inhibit the activation of the nuclear factor κB (NF-ᴋB) [50].

**Table 2:** Lipidic profile and vascular parameters in serum.

	Parameters	Group SD			Group HD			Group JP		
**Lipidic ProFile #**	Cholesterol (mg/dL)	38.66 ^a^	±	0.70	54.76 ^b^	±	2.51	54.76 ^b,c^	±	2.08
	cHDL (mg/dL)	20.68 ^a^	±	0.71	22.48 ^a^	±	1.75	28.35 ^b^	±	1.12
	cLDL (mg/dL)	10.13 ^a^	±	0.90	15.60 ^b^	±	1.91	9.54 ^a,c^	±	0.95
	Triglycerides (mg/dL)	60.44 ^a^	±	2.69	132.09 ^b^	±	3.40	142.30 ^c^	±	10.98
Vascular Markers *	E- selectin (ng/mL)	8.63 ^a^	±	0.15	11.03 ^b^	±	0.44	6.27 ^c^	±	0.04
	s-ICAM-1 (ng/mL)	7.71 ^a^	±	0.21	7.83 ^a^	±	0.24	7.36 ^a^	±	0.11
	VWF (ng/mL)	8.64 ^a^	±	0.24	9.06 ^a^	±	0.34	10.9 ^b^	±	0.49
	Adiponectin (ng/mL)	70.39 ^a^	±	2.36	58.97 ^b^	±	1.48	69.37 ^a^	±	1.17

SD: standard diet; HD: high-fat diet; JP: high-fat diet + pomegranate juice. (^a^) ^#^ Values represent the mean ± standard error. Statistical analysis for biochemical parameters was determined by using a one-way ANOVA followed by the Tukey test for multiple comparisons with a significance level of *p* ≤ 0.05. Different letters indicate statistical significance between the groups (columns). (^b^) * Values represent the mean ± standard error. Statistical analysis for vascular parameters was determined by using a one-way ANOVA followed by the Bonferroni test for multiple comparisons with a significance level of *p* ≤ 0.05. Different letters (^a^, ^b^ and ^c^) indicate statistical significance between the groups (columns).

The maximum reduction in LDL-C with statins ranged from 30% to 50%, while the combination of statins with other drugs reduced it from 60% to 84% [51]. On the other hand, the comparison in the efficacy of different doses of atorvastatin versus simvastatin, pravastatin, lovastatin, and fluvastatin in hypercholesterolemic patients showed that atorvastatin 10, 20, and 40 mg for 8 weeks reduced LDL-C by 38%, 46%, and 51%, respectively [52]. Various epidemiological studies have revealed that statins increase HDL-C by approximately 5% to 7% and that LDL-C decreases by an additional 5%–6% when the statin dose is doubled, compared to that achieved with the minimum dose of 10 mg in such a way that these increasing statin doses do not progressively increase HDL-C levels, as is the case with atorvastatin [53]. Due to the above, pomegranate juice, given the results found in this study with an increase in HDL-C levels in 27% and a reduction in LDL-C in 39%, it is likely that the effect is due to the antioxidant power and the interaction of the different components of the juice, among others, the concentration of anthocyanins, since they have been shown to reduce blood pressure and arterial stiffness [54], such that clinical studies must be carried out that provide information on the effect on cholesterol levels, compared with the total or partial replacement of atorvastatin with the advantage of not generating side effects, such as the case of statins whose main side effects are muscle damage, generation of DT2, and bleeding risk (American Heart Association). Some further comparative studies in the same models are necessary to corroborate this assessment [55].

### 3.4. Vascular Parameters

Atherosclerosis is a disease characterized by a chronical arterial wall inflammation and endothelial dysfunction, among others. In this condition, the activated endothelial cells overexpress several cell adhesion molecules, such as vascular cell adhesion molecule-1 (VCAM-1), intercellular adhesion molecule-1 (ICAM-1), platelet endothelial cell adhesion molecule (PECAM-1), and selectins (E-selectin and P-selectin) on their surfaces. Since the estimation of these molecules could represent a vascular damage marker, in this model E selectin, ICAM1, and the von Willebrand factor (VWF) were evaluated.

The results show (Table 2) that the HD group presented higher levels of selectin (11 ng/mL) than the treatment group (8.63 ng/mL), which suggests a tendency to endothelial damage induced by a high-fat diet, while the group treated with pomegranate juice had decreased sE-S levels by 42%, suggesting a regulator effect on endothelial damage; therefore, the pomegranate juice has an antiatherogenic effect. The reduction in the sE-S levels in the group treated with the juice will surely prevent damage to the vascular endothelium since the initial rolling process would not be carried out.

In relation to ICAM-1, pomegranate juice has a tendency to reduce its levels. In view of the fact that the levels of sE-S were low in the treated group, even lower levels of ICAM-1 would be expected; thus, by extending the study time, a decrease with the treatment will likely be observed.

ICAM-1 is expressed in epithelial cells, fibroblasts, macrophages, and lymphocytes in small quantities, although it is hardly expressed in the endothelium, and it only increases in cases of acute and chronic inflammation generated by TNF-α and IL-1, increasing its circulating concentration. Previous studies have shown that supplementation with epigallocatechin in patients with clinically stable coronary heart disease improves endothelial function, so the effect may be due to both the gallic acid and catechin present in the juice [54,56]. As for the VWF factor, contrary to what was expected, it increased by 20% in the group treated with pomegranate juice compared to the control group. There was a marked imbalance between the VWF and A disintegrin-like and metalloprotease with thrombospondin type 1 motif no. 13 (ADAMTS13) induced by thrombosis that is caused by inflammation. Different mediators of inflammation (Il6, Il-8, TNF-α, superoxide anions, histamine, and thrombin) produce an increase in VWF levels through various mechanisms [57]; therefore, it is probable that some of the cytokines that are elevated due to one of the different mechanisms involved increase in such a way that the compounds present in the juice cannot counteract this response. For an adequate explanation, additional studies focused on this factor are required.

The results suggest that some compounds present in the juice promote the increase in the VWF factor, in contrast to the net effect (low levels of HDL cholesterol, selectin, sICAM-1, and adiponectin) that shows an antithrombotic effect. In addition to previous evidence, pomegranate juice has a protective vascular effect; for example, in studies in mice with apolipoprotein E deficiency and supplemented with pomegranate juice, the size of the atherosclerotic lesions was reduced by 44% in addition to a decreased number of foam cells in such lesions.

Furthermore, it has been shown that the presence of pomegranate juice in the endothelial cells of the human coronary artery reverses the low regulation of nitric oxide synthase (eNOS) expression caused by the addition of oxLDL [58], probably due to the high content of antioxidant phenolic compounds, such as punicalagin, catechin, chlorogenic, gallic, ferulic, caffeic acid and ellagic acids, and anthocyanins.

Adiponectin levels (ADP) (Table 2) increased by 17% with pomegranate juice, compared to the HD group. Low levels of adiponectin are associated with a state of inflammation in the adipose tissue generated by a high-calorie diet. In this model, the JP treatment stimulated adiponectin secretion; this adipokine has antidiabetic, anti-inflammatory, and antiatherogenic effects, in addition to causing an increased expression of molecules involved in the transport of fatty acids (CD36), oxidation of fatty acids (Acyl CoA oxidase), and energy dissipation (Uncoupling protein 2, UCP-2) [59,60]. The high levels of adiponectin with the percentage of adipose tissue in the treatment group clearly denotes an inverse relationship, which helps maintain muscle and liver oxidation of fatty acids. Likewise, the levels of E-selectin, cHDL, cLDL, and blood pressure of the group treated with pomegranate juice evidence that the treatment, as shown by increased adiponectin levels, decreased cardiovascular risk factors [61,62].

### 3.5. Markers of Inflammation and Cytokines in Adipose Tissue

**Figure 1:**
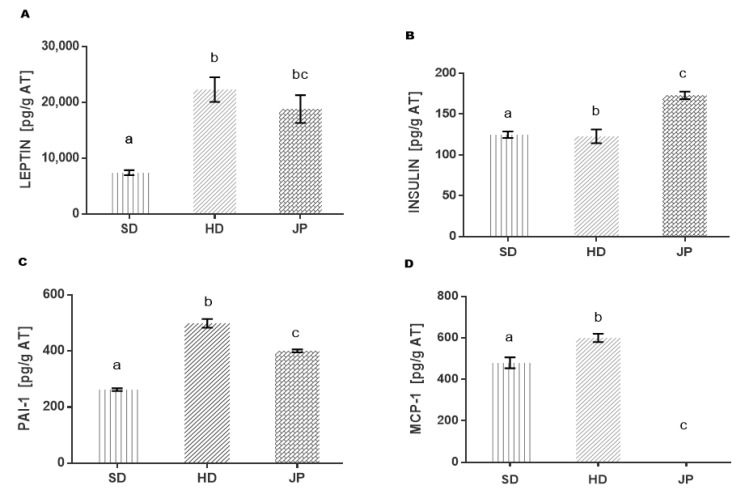
Effect of the polyphenols present in pomegranate juice on markers of inflammation in adipose tissue. (**A**) Leptin, (**B**) insulin, (**C**) plasminogen activator inhibitor-1(PAI-1), (**D**) monocyte chemoattractant protein-1(MCP-1); standard diet (SD); high-fat diet (HD); high-fat diet + pomegranate juice (JP). Statistical analysis was determined using the non-parametric Mann–Whitney–Wilcoxon test with a significance level of *p* ≤ 0.05. Different letters (^a^, ^b^ and ^c^) represent the significant statistical difference between the groups.

Since the effect of juice on inflammation markers has only been reported in serum, in this study, the effect was determined directly in retroperitoneal adipose tissue, which is metabolically more active than the epididymal tissue. The changes in the levels of leptin, insulin, MCP-1, and PAI-1 due to the administration of pomegranate juice are shown in Figure 1. The adipose tissue of the group of animals treated with JP was 15% lower compared to the adipose tissue developed in the animals of the HD group, and although there was no difference in leptin levels, adiponectin levels were increased (Figure 1A). In view of the fact that leptin is secreted by adipocytes and that plasma leptin concentration is known to be proportional to the volume of adipose tissue, it would be expected that blood leptin levels would be lower in the JP group, representing an advantage as due to this relationship, the regulation of the energy balance is promoted, thereby suppressing food intake and promoting energy expenditure according to nutritional status [63,64,65].

Insulin levels increased by 41% in the group treated with pomegranate juice compared to the HD group without insulin resistance, while the SD and HD groups did not show any difference (Figure 1B). This is important since although there is no difference in insulin levels between the SD and HD groups, the glucose levels in the HD group were increased, while in the JP group they were adequately reduced since the main function of insulin is to keep glucose levels at normal intervals and to promote its uptake in adipose tissue, muscle, and the liver. Therefore, the results of pomegranate juice suggest an efficient glucose uptake in adipose tissue, likely by some mechanism of protection of the β-pancreatic cells.

In the JP group (Figure 1C), PAI-1 decreased by 25% compared to the HD group, even though it did not reach the levels of the SD group, which suggests an anti-atherosclerotic and antithrombotic effect benefiting the cardiovascular system since high levels of PAI-1 generate the development of fibrin deposits, so that fibrin enhances fibrosis, atherogenesis, and arterial thrombosis [66].

TNF-α regulates the increase in PAI-1 in adipocytes through the TNF-α signaling pathway linked to TNFR1, which involves the activation of p42/p44, PKC, p38, PI3K, NFκB, and reactive oxygen species (ROS) production [67], increasing inflammation. 

According to the low levels of TNF-α and PAI-1 in the JP group, this relationship leads to a lower risk of vascular complications [68,69]. As is well described in various studies, MCP-1 is the monocyte chemoattractant protein with proinflammatory effects, whose levels are increased in response to tissue damage. Regarding the quantified MCP-1 levels (Figure 1D), they were high for the HD control group, whereas this marker was not detected with the pomegranate juice treatment. It is possible that the pomegranate juice could regulate the expression of the gene and consequently did not express the protein by the action of the phenolic compounds present (anthocyanins, ellagic acid); thus, it decreased the RI, the inhibition of the expression of Glucose Transporter Protein 4 (GLUT-4), Peroxisome Proliferator Activated Receptors γ (PPARγ), and the fatty acid transporter protein (FATP) [70].

There are studies with obese rodents and humans that show that circulating MCP-1, as well as the MCP-1 ligand (motif ligand 2 (part of a protein sequence that is associated with a particular biological function)) C-C, CCL2, or MCP-1), are increased in proportion to the level of obesity, and they decrease after treatment with thiazolidinediones [71,72]. Efforts have been made to inhibit MCP-1 overproduction and to reduce obesity-related comorbidities, such as insulin resistance and type 2 diabetes. In particular, some treatments of phenolic compounds have been reported to reduce MCP-1 levels, for example, 11 g of bamboo leaf ethanolic extract was used for 6 months in C57BL/6J mice, reducing the circulating MCP-1 levels by 49%. In another study, they used capsaicin (0.015%) for 10 weeks in obese mice, decreasing the expression of MCP-1 mRNA in adipose tissue.

Procyanidins extracted from grape seeds evaluated in human adipocytes (SGBS) and macrophages (THP-1) reduced the expression of MCP-1 and IL-6 by inhibiting the translocation of the NF-κB to the nucleus in both cell lines, thus improving the production of adiponectin [73,74,75,76].

In this study, proinflammatory cytokines (IL-α, IL-β, IL-6 and TNF-α) that promote insulin resistance at the post-receptor level in the adipose tissue through the PI3K pathway and are involved in both metabolic regulation and inflammatory processes were evaluated [77,78,79]. In addition, anti-inflammatory cytokines, such as interleukin 10 (IL-10) and adiponectin, were evaluated.

**Figure 2:**
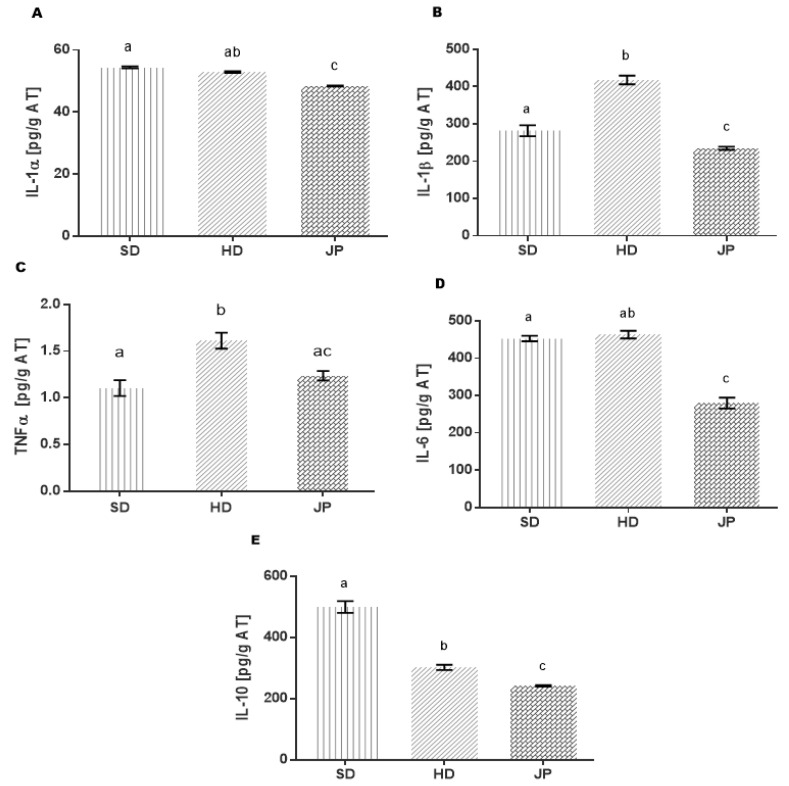
Effect of the polyphenols present in pomegranate juice on the regulation of interleukins in adipose tissue. (**A**) Interleukin 1α (IL-1α); (**B**) interleukin 1β (IL-1β); (**C**) tumor necrosis factor (TNF-α); (**D**) interleukin 6 (IL-6); (**E**) interleukin 10 (IL-10). Groups are abbreviated as standard diet (SD); high-fat diet (HD); high-fat diet + pomegranate juice (JP) group with treatment. Statistical analysis was determined by using the non-parametric Mann–Whitney–Wilcoxon test with a significance level of *p* ≤ 0.05. Different letters (^a^, ^b^ and ^c^) represent the significant statistical difference between the groups.

The results in Figure 2A,D show that in the obese group (HD) there was no change in the levels of IL-1α, and IL-6 compared to the healthy group (SD); in contrast, the levels of IL-1β and TNF-α (Figure 2B,C) in the HD group increased by 48% and 46%, respectively. In addition, the IL-10 levels (Figure 2E) decreased by 39% compared to the SD group. Deregulation of these cytokines demonstrates a state of inflammation induced by a hypercaloric diet. Since proinflammatory cytokines in adipose tissue are expressed mainly by macrophages [79], in obesity and diabetes, lipid accumulation has been reported to trigger an immune response related to macrophage activation by polarizing to an M1 profile [80].

On the other hand, the JP group that was treated with pomegranate juice had reduced IL-1α levels by 9%, IL-6 by 40%, IL-1β by 44%, and TNF-α by 25%, whose values were very similar to those of the SD group. These results show that pomegranate juice regulates the production of proinflammatory cytokines by induction, possibly in the phenotypic change of macrophages from an M1 to M2 profile [81]. The regulation of this response would be expected to be mediated by the production of anti-inflammatory cytokines, such as IL-10, which, in obesity, has elevated levels as a homeostatic mechanism in response to a state of inflammation [82,83,84]. However, IL-10 levels in the JP group decreased by 20% compared to the HD group; even though the increase was not observed in this model of IL-10, it was observed that adiponectin was increased in response to treatment. The regulation of proinflammatory cytokines in the JP group may also be associated with the effect of polyphenols (anthocyanins and ellagic acid) present in pomegranate juice by interfering with the signaling pathways that lead to the production of these cytokines, with an antagonistic effect of the receptors that signal its production and/or blocking translocation to the nucleus of transcription factors, such as nuclear factor κB (NF-kB), preventing the expression of proinflammatory genes (TNF-α, IL-6 and IL-1) [85,86].

Different studies report that polyphenols promote health through multiple signaling pathways (such as lipid anabolism/catabolism pathways and apoptotic pathways) [87].

Pomegranate juice suppresses TNF-α-induced COX-2 expression, which in turn activates the NF-κB and Akt pathway, suggesting that the anthocyanins and flavones present in pomegranate juice may be responsible [88]. The metabolites products derived from the digestion in the gastrointestinal tract of cyanidine 3-glucoside, such as protecatecuic acid, inhibit the production of IL-6, TNF-α, IL-β and prostaglandin E2 (PGE2), suppressing the nuclear factor-kB and the extracellular signal-regulated kinase (ERK) [89]. Purple corn containing cyanidin 3-glucoside (C3G) works as a powerful antioxidant in vivo (C57BL/6 male mice), whose administration with 2 g/Kg per day for 12 weeks suppresses proinflammatory cytokines as TNF-α in the acute process of inflammation [90]. On the other hand, it is likely that pomegranate juice favors autophagy, thus explaining the decrease in IL-1β since it has been shown that its production is very finely regulated by autophagy, so that molecules are negatively regulated in this way and involved in the production of IL-1β [65].

Pomegranate juice demonstrates its potential as a nutraceutical due to its complex composition of phytochemicals (hydrolysable tannins, anthocyanins, and ellagic acid) with an immunoregulatory and therapeutic effect on cardiovascular risks, maintaining normal pressure levels due to its antioxidant and protective power since it generates the balance between cHDL and cLDL. It reduces damage to the endothelium by attenuating the levels of sE-S, preventing its progression to atherosclerosis that is generated by obesity.

Likewise, it has a regulatory effect on the immune system, reducing cytokine levels and proinflammatory markers (TNF-α, IL-1α, IL-1β, IL-6) and increasing the levels of adiponectin (anti-inflammatory interleukin), in addition to being a possible MCP-1 inhibitor, promoting the good functioning of adipose tissue. Hence, we suggest the promotion of the use of pomegranate juice as an adjuvant and/or drug substitute to help improve the deteriorated state of health due to obesity-generated pathophysiology (chronic low-grade inflammation), metabolic syndrome, and type 2 diabetes. Our results demonstrate that pomegranate juice reduces the concentration of inflammation markers, the mechanisms for which this occurs are not known, so it is important to know, among others, how pomegranate juice modulates the expression of genes associated with inflammation of adipose tissue in obese. Likewise, clinical trials are needed to evaluate the effect of pomegranate juice on inflammation markers and gene expression associated with inflammation in the plasma of obese people in order to relate them to preclinical studies. It is thus convenient to evaluate the effect of pomegranate juice compared to that of statins in hypertensive people since the results of this study show a significant decrease in blood pressure. An understanding of the mechanisms by which MCP-1 inhibition occurs, as well as the increase in the von Willebrand factor is required. Although this study was conducted in an animal model, the results are promising and could be a guideline for further studies in human clinical trials to establish the potential advantage to use derivatives of pomegranate as a treatment to metabolic diseases.

## 4. Conclusions

This study provides relevant findings in the field of metabolic diseases. The hallmark of obesity is the accumulation of dysfunctional adipose tissue, which induces a plethora of complications that can cause low-grade chronic systemic inflammation. Together, these results emphasize that pomegranate juice reduces some of the major cytokines related with the low-grade inflammation and parameters associated with cardiovascular risk in obesity. Since in obesity adipose tissue is the niche for cells involved in the production of several bioactive molecules that promote chronic inflammation, a remarkable success of this work is that the effect of pomegranate juice was analyzed in this tissue. However, extensive studies are necessary in order to understand the plausible active compound involved in the positive effect of pomegranate juice, as well as the molecular mechanism implicated in the homeostatic inflammation promoted by this nutraceutical. Nevertheless, the consumption of pomegranate juice could represent a potential alternative as an adjuvant in the treatment of obesity.
